# Appointments to Utica Insane Asylum

**Published:** 1878-11

**Authors:** 


### Appointments to Utica Insane Asylum.

The Utica papers announce the appointment of Dr. E. N. Brush as 3d Assistant Physician in place of Dr. Livingston, resigned, and Dr. W. W. Miner, 4th Assistant Physician in place of Dr. Kenrick, now in Europe.

In these appointments the Buffalo Medical and Surgical Journal, as well as the citizens of Buffalo generally, suffer great loss. Their numerous friends will deeply regret their removal from this city where they have both already gained enviable reputation in the profession, and with the discriminating and intelligent public. We try to relieve our feelings of personal loss in the gratification of seeing real worth so positively and thoroughly appreciated and rewarded, for certainly the Medical Institutions of the State do not offer more honorable or desirable position for physicians, and we most heartily congratulate them upon the opportunity thus opened up to them.

